# Association of Pulmonary Sepsis and Immune Checkpoint Inhibitors: A Pharmacovigilance Study

**DOI:** 10.3390/cancers15010240

**Published:** 2022-12-30

**Authors:** Shuang Xia, Hui Gong, Yichang Zhao, Lin Guo, Yikun Wang, Bikui Zhang, Mayur Sarangdhar, Yoshihiro Noguchi, Miao Yan

**Affiliations:** 1 Department of Pharmacy, The Second Xiangya Hospital, Central South University, Changsha 410011, China; 2 International Research Center for Precision Medicine, Transformative Technology and Software Services, Changsha 410011, China; 3 Toxicology Counseling Center of Hunan Province (TCCH), Changsha 410011, China; 4 Division of Biomedical Informatics, Cincinnati Children’s Hospital Medical Center, Cincinnati, OH 45229, USA; 5 Division of Oncology, Cincinnati Children’s Hospital Medical Center, Cincinnati, OH 45229, USA; 6 Department of Pediatrics, University of Cincinnati College of Medicine, Cincinnati, OH 45229, USA; 7 Laboratory of Clinical Pharmacy, Gifu Pharmaceutical University, Gifu 501-1196, Japan

**Keywords:** immune checkpoint inhibitors, pulmonary sepsis, pharmacovigilance, FAERS, disproportionality analysis

## Abstract

**Simple Summary:**

This study investigates the association of pulmonary sepsis with immune checkpoint inhibitors by conducting an analysis using data from the Food and Drug Administration pharmacovigilance database. Compared to chemotherapy or targeted therapy, a robust signal emerged for nivolumab and atezolizumab. Co-administration of immune checkpoint inhibitors and glucocorticoids or proton pump inhibitors synergistically increased the risk of pulmonary sepsis. These signals should promote both prospective research and multidisciplinary proactive monitoring by healthcare professionals.

**Abstract:**

Background: Although some sepsis cases were reported with immune checkpoint inhibitors (ICIs) in clinical trials, the link between pulmonary sepsis and ICIs remains mostly unknown. We aim to investigate the association between pulmonary sepsis and ICIs, and to describe the clinical features. Methods: A disproportionality analysis was performed using FAERS data and compared rates of pulmonary sepsis in cancer patients receiving ICIs vs. other drug regimens (such as chemotherapy and targeted therapy). Associations between ICIs and sepsis were assessed using reporting odds ratios (ROR) and information component (IC). We also detected drug interaction signals based on the Ω shrinkage measure. Age and gender distribution were compared between pulmonary sepsis and all adverse events associated with ICIs. Results: We identified 120 reports of pulmonary sepsis associated with ICIs between Q1, 2011 to Q3, 2021. A total of 82 of 120 (68.3%) patients on ICIs suffered from pulmonary sepsis and progressed to death. In addition, there is no significant difference in age and gender in the occurrence of pulmonary sepsis in cancer patients on ICIs. Overall ICIs, nivolumab, and atezolizumab still have a significant signal of pulmonary sepsis (ROR_025_ > 1, IC_025_ > 0, *p* < 0.001) compared with targeted therapy (such as tyrosine kinase inhibitors) or chemotherapy. Co-administration of ICIs and glucocorticoids or proton pump inhibitors synergistically increased the risk of pulmonary sepsis (Ω_025_ > 0). Conclusions: Our study suggested ICIs, especially nivolumab and atezolizumab, tended to increase the risk of pulmonary sepsis more than other anticancer regimens. Clinicians should be vigilant in the prevention and management of pulmonary sepsis during ICIs therapy.

## 1. Introduction

Immune checkpoint inhibitors (ICIs) have transformed the treatment landscape of numerous cancers, generating durable responses in many patients [1]. Programmed cell death 1 (PD-1) and cytotoxic T lymphocyte antigen 4 (CTLA-4) are co-inhibitory receptors expressed on the surface of T cells to negatively regulate T cell-mediated immune responses; however, tumor cells exploit these inhibitory molecules to induce tumor tolerance and T cell exhaustion. Accordingly, ICIs such as anti-CTLA-4, anti-PD-1, and anti-PD-L1 can attach to these co-inhibitory receptors, thereby reactivating the immune response against tumor cells [2]. LAG-3 is a transmembrane protein involved in cytokine release and inhibitory signaling in T cells. Preclinical data showed that LAG-3 is a negative regulator of both the CD4 T cell and CD8 T cell and the activity on the CD8 T cell is independent of CD4 activation. On the CD8 T cell, LAG-3 activation abrogates the antigen presentation, whereas arrests the S phase of the cell cycle on the CD4 T cell. Based on that, the inhibition of LAG-3 is relevant and could have promising clinical benefits in treating several solid tumors [3]. The FDA approved ICI regimens including PD-1 inhibitors: nivolumab, pembrolizumab, cemiplimab, dostarlimab; PD-L1 inhibitors: atezolizumab, avelumab, durvalumab; CTLA-4 inhibitors: ipilimumab, tremelimumab; LAG-3 inhibitors: relatlimab; and combination therapy of ICIs (ipilimumab and nivolumab). Toxic effects from these ICIs agents are related to removing nodes of self-tolerance and unleashing autoimmune-like phenomena [4]. Although usually manageable with corticosteroid and immunosuppressants administration, clinically severe events leading to morbidity and even mortality may complicate ICIs treatment [5]. 

Sepsis is a life-threatening organ dysfunction resulting from dysregulated host responses to infection [6]. The Sequential Organ Failure Assessment (SOFA) score is used to codify the degree of organ dysfunction [7]. Sepsis is a common condition that is associated with disproportionately high mortality and, for many of those who survive, long-term morbidity. The World Health Organization (WHO) made sepsis a global health priority in 2017 and has adopted a resolution to improve the prevention, diagnosis, and management of sepsis [8]. A recent study [9] showed that cancer patients with sepsis have a higher mortality rate than non-cancer patients. During sepsis, the primary site of infection is the lung (67.4%), followed by the abdomen (20%) [10]. Pulmonary sepsis is usually characterized by hypoxemia and impaired gas exchange. It is generally referred to as acute lung injury (ALI), which further results in acute respiratory distress syndrome (ARDS), the severe form [11]. A recent meta-analysis [12] identified five cases of sepsis, ranked the second reason for fatal adverse events associated with PD-L1 inhibitors. Another case [13] reported grade 5 sepsis occurred with pembrolizumab (PD-1 inhibitor) and caused death in the KEYNOTE-028 study. The data from clinical trials with strict inclusion criteria and cohorts with limited sample sizes may not sufficiently represent the real clinical setting. In addition, there is no study that analyzed the link between pulmonary sepsis and immune checkpoint inhibitors. 

Given the widespread use of ICIs in clinical practice and the potentially life-threatening nature of sepsis, it is critical for clinicians to realize the safety concern and clinical manifestations of sepsis correlated with ICIs. This pharmacovigilance study aims to investigate the potential association between pulmonary sepsis and ICIs and characterize the main features of pulmonary sepsis with ICIs in the FAERS database.

## 2. Methods

### 2.1. Study Design and Data Sources

This retrospective pharmacovigilance study is a disproportionality analysis based on deidentified individual case safety reports (ICSRs) in FAERS, the FDA’s Adverse Events Reporting System, which allows for the signal detection and quantification of the association between drugs and reporting of AEs (adverse effects). We used AERS*Mine* [14], a validated web-based platform that analyzes FAERS reports for AEs association with drugs, indications, demographics (age and gender), and reporters. Several studies [15,16] have used AERS*Mine* to analyze FAERS data, including a recent study that combined clinical cardiotoxicity of kinase inhibitors with cell line-derived transcriptomic datasets to identify a gene signature that can predict the risk of cardiotoxicity [17]. Ethical approval was not required because this study was conducted by using deidentified data.

### 2.2. Procedures

A pharmacovigilance study was conducted from 2011 Q1 (because ipilimumab was the first ICI approved by FDA on 25 March 2011) to 2021 Q3 with the FAERS data in AERS*Mine* to evaluate the risk of pulmonary sepsis correlated with ICIs in a large-scale population. We included eight FDA-approved ICI regents (nivolumab, pembrolizumab, ipilimumab, atezolizumab, avelumab, durvalumab, dostarlimab, cemiplimab, because tremelimumab and relatlimab were recently approved by the FDA and cases were scarce) and one ICI combination therapy (nivolumab plus ipilimumab). Firstly, we analyzed the signals of all PT (preferred terms) under sepsis (SMQ, Standardized MedDRA Query, narrow) according to the Medical Dictionary for Regulatory Activities (MedDRA 25.0). Detailed PT terms could be found in supplementary file S1. Only case numbers of more than five were included in this study. We used case/non-case analysis to analyze if sepsis was differentially reported with ICIs as compared to other drugs in the full database. Then, we selected the preferred term under sepsis (SMQ) with the strongest signal and displayed detailed clinical characteristics, which included age, gender, indication, outcome and co-treatment drugs.

To assess the robustness of disproportionality signals between immune checkpoint inhibition and sepsis and account for underlying confounders of the drug-event association, we selected the preferred term under sepsis (SMQ) with the strongest signal, and then compared the safety signal among ICIs and other traditional cancer regimens, such as chemotherapy and targeted therapy, as a comparator (to reduce confounding by indication and provide a clinical perspective). First, we identified relevant NCCN (National Comprehensive Cancer Network) guidelines (list can be found in supplementary file S1) according to FDA-approved indications of ICIs. Then we extracted chemotherapy and targeted therapy from those selected NCCN guidelines. AERS*Mine* was used to analyze safety signal variation among different regimens. Previous studies [18,19] showed that concomitant medications, such as steroids, proton pump inhibitors (PPI), and antibiotics, might affect clinical outcomes with immune checkpoint inhibitors. We inferred that the drug–drug interaction (DDI) between ICIs and these aforementioned drugs may also affect sepsis safety signals. Therefore, we conducted signal detection for drug–drug interaction between ICIs and other adjuvant drugs.

### 2.3. Statistical Analysis

In this study, safety signals were used as indicators of disproportionality in the reporting odds ratio (ROR) [20] based on the frequentist statistical method; and the information component (IC) [21] based on the Bayesian statistical method used at the Uppsala Monitoring Centre (UMC). The detection criterion was the lower limit of the 95% confidence interval (CI) of ROR(ROR_025_) > 1 [20], and the lower limit of the 95% confidence interval of IC (IC_025_) > 0 [21]. Norén et al. [22] put forth shrinkage observed-to-expected ratios, which added effective protection against spurious associations in signal detection. This IC and ROR approach has recently been proven effective to characterize the spectrum and characteristics of neurologic toxicity of checkpoint inhibitors [23]. Many algorithms [24] have also been reported to search for drug–drug interaction (DDI) signals. Among them, the Ω shrinkage [25] measure used by the UMC [26] has shown that it has the most conservative detection trend in the previous study [27]. The detection criterion is the lower limit of the 95% confidence interval of the Ω (Ω_025_) > 0. The detailed calculation process of ROR, IC, and Ω can be found in supplementary file S1. Differences in categorical variables were assessed using a chi-squared test of independence performed on a 2 × 2 contingency table with Yates’ continuity correction or Fisher’s exact test. Significance was assumed when the *p* value was less than 0.05. All data analyses were performed independently by two authors and statistical analyses and calculations were performed with IBM SPSS Statistics for Windows, Version 26.0. Armonk, NY: (IBM, Chicago, IL, USA), and Microsoft Excel 2021 (Microsoft Corporation, Redmond, WA, USA).

## 3. Results

### 3.1. Sepsis Signal Detected Using FAERS Database

From a total number of 215,363 case reports of patients on ICIs in the full FAERS between 2011 (Q1) and 2021 (Q3), we detected 7535 cases of PT terms under sepsis (SMQ). Compared with all other drugs in the FAERS database, ICIs have a significant safety signal of sepsis (SMQ) (ROR_025_ 2.72, IC_025_ 1.37). Moreover, the pulmonary sepsis attributes the strongest signal (ROR_025_ 6.43, IC_025_ 2.48) among all detected PT terms under sepsis (SMQ). Sepsis toxicities associated with immune checkpoint inhibitors are detailed in Table 1.

**Figure 1:**
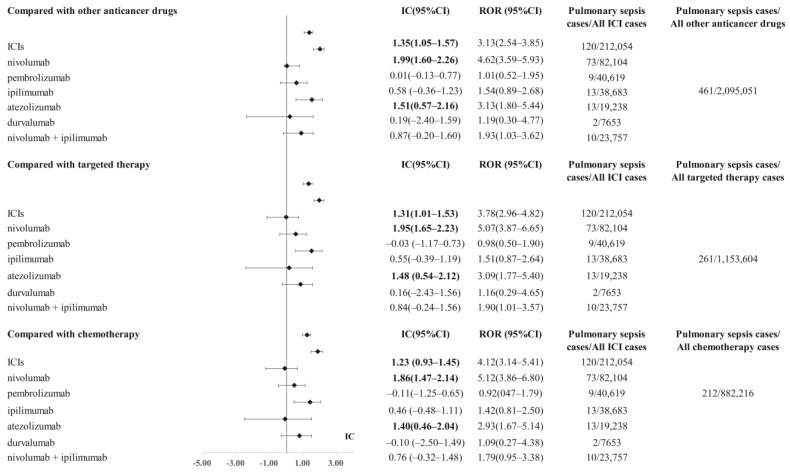
The comparison of pulmonary sepsis signal between ICIs and controls (other anticancer drugs, targeted therapy, chemotherapy) in FAERS database. The list of the control group (targeted therapy and chemotherapy) was extracted from NCCN guidelines for ICIs’ indication. More details could be found in the AERS*Mine* load files in the supplementary file S3. Abbreviation: ROR, reporting odds ratio. IC, information component. 95%CI, 95% confidence interval. N, number. AEs, adverse events. ICIs, immune checkpoint inhibitors. NCCN, National Comprehensive Cancer Network.

Then, we assessed the robustness of the association of pulmonary sepsis with ICIs (nivolumab, pembrolizumab, ipilimumab, atezolizumab, durvalumab, nivolumab plus ipilimumab were included in the subsequent analysis because the case number of dostarlimab, avelumab, and cemiplimab is less than 5). When compared with other anti-cancer drugs, overall ICIs (ROR_025_ 2.50, IC_025_ 1.03, *p* < 0.001), nivolumab (ROR_025_ 3.59, IC_025_ 1.60, *p* < 0.001), atezolizumab (ROR_025_ 1.80, IC_025_ 0.57, *p* < 0.001) showed a significant safety concern of pulmonary sepsis. When compared with targeted therapy (such as tyrosine kinase inhibitors) or chemotherapy extracted from NCCN’s guideline, overall ICIs, nivolumab, and atezolizumab still have a significant signal of pulmonary sepsis (ROR_025_ > 1, IC_025_ > 0, *p* < 0.001) (Figure 1).

### 3.2. Clinical Features of Pulmonary Sepsis

**Figure 2:**
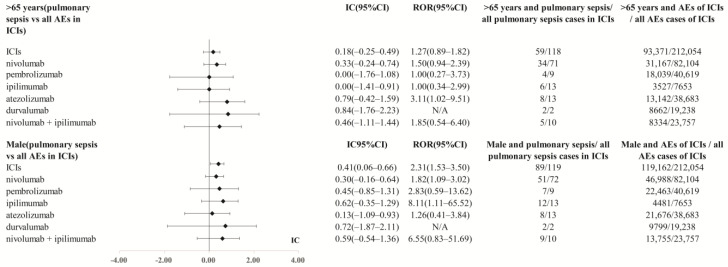
Age and gender distribution in pulmonary sepsis cases of ICIs. ROR, reporting odds ratio. IC, information component. 95%CI, 95% confidence interval. AEs, adverse events. ICIs, immune checkpoint inhibitors. N/A, not applicable. (Because the case number < 3, ROR could not be calculated).

120 cases of pulmonary sepsis were detected in patients on ICIs. A total of 73 of 120 (60.8%) cases were from patients on nivolumab. A total of 85.8% of cases were reported during 2018–2021. A total of 83 of 120 (69.2%) pulmonary sepsis cases were reported by health professionals such as doctors or pharmacists. Regarding indications, 40 of 120 (33.3%) cases were reported as lung cancer. A total of 48 of 120 (40.0%) pulmonary sepsis cases received ICIs and glucocorticoids/corticosteroids, and 44 of 120 (36.7%) cases received proton pump inhibitors during ICIs therapies (Table 2). After comparing the age of pulmonary sepsis (age of more than 65) associated with ICIs against the same age range of any reported adverse events of this drug class, we did not detect any difference of signals among various age periods. Although, the overall ICIs (IC_025_ 0.06, *p* < 0.001) showed that males have a higher safety concern of pulmonary sepsis than females. However, we did not find any difference of pulmonary sepsis signals between males and females for any specific kind of ICIs (Figure 2). A total of 82 of 120 (68.3%) cases suffering from pulmonary sepsis progressed to death for patients on ICIs. A total of 31 of 120 (25.8%) cases experienced a life-threatening situation.

### 3.3. Drug-Drug Interaction Signal Detection

We identified that co-administration of nivolumab (Ω_025_ = 0.91), ipilimumab (Ω_025_ = 0.77), nivolumab plus ipilimumab (Ω_025_ = 1.04) with glucocorticoids or corticosteroids have an elevated safety concern of pulmonary sepsis. In addition, the co-treatment of nivolumab (Ω_025_ = 1.00), nivolumab plus ipilimumab (Ω_025_ = 0.17) with proton pump inhibitors may also synergistically increase the risk of pulmonary sepsis (Table 3).

When Ω_025_ > 0, a significant drug–drug interaction signal was detected. The detailed calculation process of Ω_025_ can be found in supplementary file S1 and raw data could be found in supplementary file S2. 

## 4. Discussion

To the best of our knowledge, this is the first large-scale pharmacovigilance study on pulmonary sepsis associated with ICIs leveraging the FAERS database. In general, there were three key findings in our study. Firstly, our disproportionality analyses suggested ICIs may increase the risk of pulmonary sepsis compared with other anti-cancer agents such as chemotherapy or targeted therapy. Secondly, we investigated the detailed safety profile and clinical features of pulmonary sepsis. Finally, we identified the potential medications which would increase the risk of pulmonary sepsis when co-administrated with ICIs through drug–drug interaction signals detection. 

In this study, our analysis showed a significant incidence of pulmonary sepsis associated with ICIs, suggesting that pulmonary sepsis may be underrepresented in the published literature. As of 28 November 2022, no peer-reviewed observational studies, meta-analysis, or reviews were published related to the underlying association between ICIs and pulmonary sepsis. Although, previous clinical trial data [12] showed that sepsis is the second main reason leading to death in patients who received PD-L1 inhibitors, sepsis is not on the current NCCN guidelines for ICIs-related toxicity management [28]. By conducting a retrospective large-scale pharmacovigilance analysis, we detected that ICIs (case), especially nivolumab and atezolizumab, tended to increase the risk of pulmonary sepsis compared with non-case (other anticancer regimens included chemotherapy and targeted therapy).

Few pieces of literature demonstrated the clinical features of pulmonary sepsis associated with ICIs. We found 85.8% of pulmonary sepsis cases were reported since 2018, which reflected the accompanying safety concern of the increasing application of immune checkpoint inhibitors in cancers. A total of 25.8% of cases experience a life-threatening situation when pulmonary sepsis occurred and 68.3% of cases are finally deceased, indicating the severe outcome of this type of toxicity. A total of 60.8% of pulmonary sepsis cases were from patients on nivolumab. In addition, a study [29] showed that 62.5% of tuberculosis and 69.3% of atypical mycobacterial infections were induced by nivolumab. Our post-marketing large-scale pharmacovigilance analysis supports that PD-1 inhibitors nivolumab increase the incidence of pulmonary sepsis. We did not detect a significant difference in age and gender in the occurrence of pulmonary sepsis in cancer patients on ICIs. Further research is warranted to investigate the influence of age and gender on the occurrence of pulmonary sepsis associated with ICIs.

We identified 40.0% and 36.7% of patients suffered from pulmonary sepsis received glucocorticoids/corticosteroids or proton pump inhibitors when they were on ICIs, respectively, indicating the underlying influence of polypharmacy on the reported frequency of pulmonary sepsis. A large multicenter integrated analysis [18] showed that baseline steroids, systemic antibiotics, and proton pump inhibitors were associated with worse clinical outcomes in patients receiving ICIs. We further detected drug–drug interaction signals between ICIs and the aforementioned adjuvant medications. Our results indicated that co-administration of ICIs and glucocorticoids or corticosteroids increased the pulmonary sepsis report frequency in patients on nivolumab, ipilimumab, or nivolumab plus ipilimumab, which was in line with previous studies [30,31]. It is interesting that interaction signals of nivolumab, nivolumab plus ipilimumab, and proton pump inhibitors were detected (Table 3). Several previous studies [32,33] suggest that proton pump inhibitors negatively influence the magnitude of ICI efficacy and may increase the risk of death, which maybe results from severe alterations to the gut microbiome because of long-term use of PPIs. Regarding to the influence of proton pump inhibitors on the occurrence of pulmonary sepsis, we believe there are some possible reasons. First, proton pump inhibitors changes gut microbiota and subsequently increases sepsis susceptibility by following underlying mechanism: allowing for expansion of pathogenic intestinal bacteria, priming the immune system for a robust pro-inflammatory response, and decreasing production of beneficial microbial products such as short-chain fatty acids [34]. Second, proton pump inhibitors change upper gastrointestinal environment allows colonization of the oropharynx by gastrointestinal bacteria, which could increase the risk of pneumonia and even progress to sepsis [35]. Our study is the first to highlight that the combination of PPIs and ICIs not only negatively affects efficacy but also can increase rates of pulmonary sepsis. Therefore, physicians need to use PPIs carefully when patients are receiving ICIs therapy.

Regarding the potential mechanism of pulmonary sepsis associated with ICIs, bacteria are the pathogens most associated with pulmonary infection and sepsis; however, fungi viruses can also act as a source of infection [11]. A previous review [36] summarized that there are two main reasons for infection events associated with immune checkpoint inhibitors: one is opportunistic infections associated with immune-related adverse events treatment (such as glucocorticoids, corticosteroids or immunosuppressants); the other one is infections due to dysregulated immunity, such as cases of atypical mycobacterium infection following PD-1/PD-L1 immunotherapy in the absence of immunosuppression [37]. In addition, those infection events that occurred resulted from the aforementioned two pathways, which may progress to pulmonary sepsis without appropriate management and treatment.

Our pharmacovigilance analysis showed an increased reporting frequency of pulmonary sepsis associated with immune checkpoint inhibitors. Further preclinical and clinical studies are warranted to validate our results and confirm the link between pulmonary sepsis and ICIs.

## 5. Limitations

There are several limitations of the study that are intrinsic to FAERS [38]. First, adverse event reporting is voluntary and comes from heterogeneous sources, thus raising the possibility of incomplete information or underreporting. However, cases in the FAERS database cover many countries in the world, thus ensuring an unparalleled global assessment in diverse clinical settings. Second, detailed clinical information and diagnostic criteria are unavailable, thus limiting our assessment to those reports. Third, we are unable to definitively determine the incidence of each event using FAERS and only generate hypotheses. As with other pharmacovigilance studies, this study allows for signal detection and generation in a large population, which will need prospective and long-term validation of findings.

## 6. Conclusions

This real-world pharmacovigilance analysis of the FAERS database first identified that pulmonary sepsis was significantly associated with nivolumab and atezolizumab. In addition, when ICIs were co-administrated with glucocorticoids or proton pump inhibitors, the safety concern of pulmonary sepsis increased. Further studies need to be conducted to confirm the association, explore the underlying mechanisms, and address management strategies for pulmonary sepsis.

## Figures and Tables

**Table 1:** Checkpoint inhibitor-related sepsis reported with ICIs versus those reported in the full database from FAERS, from Q1, 2011 to Q3, 2021.

Categories	Overall ICIs	Full Database (Starting Q1,2011)	ROR (95%CI)	IC (95%CI)
Total number of ICSRs available	215,363	13,943,677		
Number of ICSRs by Sepsis subgroups				
Sepsis (SMQ)	7535	184,080	2.78 (2.72–2.85)	1.41 (1.37–1.43)
Sepsis	3599	82,046	2.96 (2.86–3.06)	1.51 (1.45–1.55)
Multiple organ dysfunction syndrome	1094	31,510	2.30 (2.16–2.44)	1.17 (1.07–1.24)
Bacteraemia	312	7850	2.64 (2.36–2.96)	1.36 (1.17–1.50)
Systemic inflammatory response syndrome	252	3189	5.47 (4.81–6.23)	2.34 (2.14–2.49)
Urosepsis	277	7014	2.62 (2.33–2.96)	1.35 (1.15–1.49)
Neutropenic sepsis	242	5447	2.97 (2.61–3.37)	1.52 (1.31–1.67)
Pulmonary sepsis	120	1105	7.77 (6.43–9.39)	2.78 (2.48–3.00)
Escherichia sepsis	74	2123	2.30 (1.83–2.90)	1.16 (0.78–1.44)
Escherichia bacteraemia	59	1456	2.69 (2.08–3.49)	1.37 (0.94–1.68)
Systemic candida	55	1466	2.49 (1.90–3.25)	1.26 (0.81–1.58)
Device related sepsis	46	1467	2.06 (1.54–2.77)	1.01 (0.52–1.36)
Staphylococcal sepsis	96	3415	1.84 (1.51–2.26)	0.86 (0.52–1.10)
Abdominal sepsis	29	637	3.04 (2.09–4.41)	1.51 (0.89–1.95)
Post-procedural sepsis	13	464	1.84 (1.06–3.19)	0.82 (−0.12–1.46)
Streptococcal sepsis	26	791	2.17 (1.47–3.20)	1.06 (0.40–1.52)
Procalcitonin increased	39	753	3.48 (2.52–4.81)	1.70 (1.17–2.08)
Cytomegalovirus viraemia	26	2448	0.68 (0.46–1.01)	−0.53 (−1.19−(−0.07))
Blood culture positive	12	1312	0.59 (0.33–1.04)	−0.73 (−1.71−(−0.06))

ICIs refer to any ICSRs reported for treatment with nivolumab, pembrolizumab, atezolizumab, avelumab, durvalumab, cemiplimab, dostarlimab, ipilimumab, and their combination. Sepsis subgroups were extracted from MedDRA 25.0, sepsis (SMQ). When IC_025_ value (>0) or ROR_025_ value (>1), a significant signal of drug-AE was detected. ICSRs, individual case safety reports. ICIs, immune checkpoint inhibitors. IC, information component. IC_025_ = lower end of a 95% confidence interval for the IC.

**Table 2:** Patient characteristics of pulmonary sepsis reports with immune checkpoint inhibitors in FAERS database.

Categories	Nivolumab	Pembrolizumab	Atezolizumab	Durvalumab	Ipilimumab	Nivolumab + Ipilimumab	All ICIs
Reports of Pulmonary sepsis	73	9	13	2	13	10	120
Report Year							
2011–2017	9 (12.3%)	2 (22.2%)	2 (15.4%)	2 (100.0%)	2 (15.4%)	0	17 (14.2%)
2018–2021 (Q3)	64 (87.7%)	7 (77.8%)	11 (84.6%)	0	11 (84.6%)	10 (100.0%)	103 (85.8%)
Reporter							
Healthcare professionals	51 (69.9%) 22 (31.1%)	5 (55.6%) 4 (44.4%)	13 (100.0%) 0	2 (100.0%) 0	7 (53.8%) 6 (46.2%)	9 (90.0%) 1 (10.0%)	83 (69.2%) 37 (30.8%)
Other							
Age Category							
0–14	0	0	0	0	0	0	0
15–24	2 (2.8%)	0	0	0	0	0	2 (1.7%)
25–65	35 (49.3%)	5 (55.6%)	5 (38.5%)	0	7 (53.8%)	5 (50.0%)	57 (48.3%)
>65	34 (47.9%)	4 (44.4%)	8 (61.5%)	2 (100.0%)	6 (46.2%)	5 (50.0%)	59 (50.0%)
Data available	71 (97.3%)	9 (100.0%)	13 (100.0%)	2 (100.0%)	13 (100.0%)	10 (100.0%)	118 (98.3%)
Gender							
Male	51 (70.8%)	7 (77.8%)	8 (61.5%)	2 (100.0%)	12 (92.3%)	9 (90.0%)	89 (74.8%)
Female	21 (29.2%)	2 (22.2%)	5 (38.5)	0	1 (7.7%)	1 (10.0%)	30 (25.2%)
Data available	72 (98.6%)	9 (100.0%)	13 (100.0%)	2 (100.0%)	13 (100.0%)	10 (100.0%)	119 (99.2%)
Indication							
Non-small Lung cancer	17 (23.3%)	5 (55.6%)	2 (15.4%)	1 (50.0%)	0	0	25 (19.2%)
Lung neoplasm malignant	14 (19.2%)	1 (11.1%)	0	0	0	0	15 (12.5%)
Other	42 (57.5%)	3 (33.3%)	11 (84.6%)	1 (50.0%)	13 (100.0%)	10 (100.0%)	80 (66.7%)
Co-administration drugs Glucocorticoids or corticosteroids	28 (38.4%)	0	1 (7.7%)	2 (100.0%)	9 (69.2%)	8 (80.0%)	48 (40.0%)
proton pump inhibitors	26 (35.6%)	2 (22.2%)	4 (92.3%)	2 (100.0%)	5 (38.5%)	5 (50.0%)	44 (36.7%)
Outcome							
Death	49 (67.1%)	6 (66.7%)	10 (76.9%)	2 (100.0%)	9 (69.2%)	6 (60.0%)	82 (68.3%)
Life-threatening	15 (20.5%)	2 (22.2%)	2 (15.4%)	1 (50.0%)	6 (46.2%)	5 (50.0%)	31 (25.8%)
Disability	1 (1.4%)	0	0	0	0	0	1 (0.8%)
Hospitalization	64 (87.7%)	9 (100.0%)	13 (100.0%)	2 (100.0%)	10 (76.9%)	8 (80.0%)	106 (88.3%)
Other Serious	71 (97.3%)	6 (66.7%)	1 (7.7%)	0	11 (84.6%)	10 (100.0%)	99 (82.5%)

We included N > 5 case reports of pulmonary sepsis related to immune checkpoint inhibitors. ICIs, immune checkpoint inhibitors. Q3, quarter 3. Glucocorticoids or corticosteroids including “dexamethasone” “prednisone” “hydrocortisone” etc.Proton pump inhibitors including omeprazole, lansoprazole, esomeprazole etc. A detailed list of included drugs can be found in the AERS load files in the supplementary file S3. Note regarding patient counts—for example, the total number of outcomes for nivolumab is not equal to total drug events (73) since patients have reported more than one outcome.

**Table 3:** Drug–Drug interaction between ICIs and other drugs.

Drug 1	Drug 2	AEs	Ω (95%CI)
Nivolumab	Glucocorticoids or corticosteroids	Pulmonary sepsis	1.45 (0.91–1.98)
Ipilimumab	Glucocorticoids or corticosteroids	Pulmonary sepsis	1.72 (0.77–2.66)
Nivolumab plus ipilimumab	Glucocorticoids or corticosteroids	Pulmonary sepsis	2.04 (1.04–3.04)
Nivolumab	Proton pump inhibitors	Pulmonary sepsis	1.55 (1.00–2.11)
Nivolumab plus ipilimumab	Proton pump inhibitors	Pulmonary sepsis	1.44 (0.17–2.70)

## Data Availability

The datasets analyzed during the current study are available in the following resource, which is available in the public domain: https://research.cchmc.org/aers/ (accessed on 26th November 2022) (AERS*Mine*, a multi-cohort analyzing application designed to mine data across millions of patient reports (currently 16,849,672) from the FDA’s Adverse Event Reporting System).

## Supplementary Materials

The following supporting information can be downloaded at: https://www.mdpi.com/article/10.3390/cancers15010240/s1, supplementary file S1: Calculation formula, preferred terms and NCCN guidelines; supplementary file S2: drug interaction ICIs and other drugs; supplementary file S3: AERS*Mine* load files.
