# Supplementary material for: Association of Pulmonary Sepsis and Immune Checkpoint Inhibitors: A Pharmacovigilance Study
Source: Cancers (Basel). 2022 Dec 30;15(1):240. doi: 10.3390/cancers15010240 (PMC9818197; doi:10.3390/cancers15010240)
Supplement: Supplementary file 1 [file cancers-15-00240-s001.zip › Supplementary file 1 Calculation formula, Preferred terms and NCCN guidelines .pdf]

cancers MDPI

Association of Pulmonary Sepsis and Immune Checkpoint Inhibitors: A Pharmacovigilance Study

Supplementary File 1

Contents:

1. Calculation method details of ROR, IC and Ω
2. Preferred terms under Sepsis (SMQ)-narrow
3. NCCN guidelines related to ICIs' indications in this study

Part one: Calculation method details in this pharmacovigilance study

Calculation method of ROR, IC is as following:

Table S1. contingency table for signal detection.

	Targeted AEs	Other AEs	Total
Targeted drugs	N_{11}	N_{10}	N_{1+}
Other drugs	N_{01}	N_{00}	N_{0+}
Total	N_{+1}	N_{+0}	N_{++}

$$ROR = \frac{(N_{11} * N_{00})}{(N_{01} * N_{10})} \quad (S1)$$

$$\text{IC (95\%confidence interval)} = e^{\ln(ROR) \pm 1.96\sqrt{\left(\frac{1}{N_{11}}+\frac{1}{N_{10}}+\frac{1}{N_{01}}+\frac{1}{N_{00}}\right)}} \quad (S2)$$

The statistical formula is as follows to calculate IC,

$$IC = \log_2\left(\frac{N_{observed} + 0.5}{N_{expected} + 0.5}\right) \quad (S3)$$

$$IC = \frac{(N_{drug} * N_{effect})}{N_{total}} \quad (S4)$$

$$IC_{025} = \log_2\left(\frac{N_{observed} + 0.5}{N_{expected} + 0.5}\right) - 3.3 * (N_{observed} + 0.5)^{-\frac{1}{2}} - 2 * (N_{observed} + 0.5)^{-\frac{3}{2}} \quad (S5)$$

$$IC_{975} = \log_2\left(\frac{N_{observed} + 0.5}{N_{expected} + 0.5}\right) + 2.4 * (N_{observed} + 0.5)^{-\frac{1}{2}} - 0.5 * (N_{observed} + 0.5)^{-\frac{3}{2}} \quad (S6)$$

$N_{expected}$: the number of case reports expected for the drug-adverse effect combination.

$N_{observed}$: the actual number of case reports for the drug- adverse effect combination.

N_{drug}: the number of case reports for the drug, regardless of adverse effects.

N_{effect}: the number of case reports for the adverse effect, regardless of the drug.

N_{total}: the total number of case reports in the database.

Calculation of drug-drug interaction signal, Ω, is as following:

Table S2. The 4 × 2 contingency table for signal detection of drug-drug interaction.

	Target AE	Other AEs	Total
**Concomitant use of drug D1 and drug D2**	n111	n110	n11+
**drug D1 without drug D2**	n101	n100	n10+
**drug D2 without drug D1**	n011	n010	n01+
**Neither drug D1 nor drug D2**	n001	n000	n00+
**Total**	n++1	n++0	n+++

AE: adverse event, *n*: the number of reports.

$$\Omega = log_2 \frac{n_{111} + 0.5}{E_{111} + 0.5} \quad \ldots (S7)$$

$$f_{00} = \frac{n_{001}}{n_{00+}},\ f_{10} = \frac{n_{101}}{n_{10+}},\ f_{01} = \frac{n_{011}}{n_{01+}},\ f_{11} = \frac{n_{111}}{n_{11+}} \quad \ldots (S8)$$

Where, *n* is the number of reports shown in the 4 × 2 contingency table.

$$g_{11} = 1 - \frac{1}{\max\left(\frac{f_{00}}{1-f_{00}}, \frac{f_{10}}{1-f_{10}}\right) + \max\left(\frac{f_{00}}{1-f_{00}}, \frac{f_{01}}{1-f_{01}}\right) - \frac{f_{00}}{1-f_{00}} + 1} \quad \ldots (S9)$$

When $f_{10} < f_{00}$ (which denote no risk of AE caused by *drug* D_1), the most sensible estimator g_{11} = max (f_{00}, f_{01}) is yielded and the *vice versa* when $f_{01} < f_{00}$.

$$E_{111} = g_{11} \times n_{11+} \quad \ldots (10)$$

$$\text{Var}(\Omega_0) = \text{Var}\left(log_2 \frac{n_{111}}{E_{111}}\right) \approx \frac{1}{n_{111} \log(2)^2} \quad \ldots (S10)$$

Where, n_{111} is the number of reports and E_{111} is the expected value.

$$\Omega_{025} = \Omega - \frac{\phi(0.975)}{\ln(2)\sqrt{n_{111}}} \quad \dots(S11)$$

Where, ϕ (0.975) is 97.5% of the standard normal distribution.

Part two Preferred terms under Sepsis (SMQ)-narrow

Abdominal sepsis; Acinetobacter sepsis; Actinomycotic sepsis; Amniotic infection syndrome of Blane; Anthrax sepsis; Bacterial sepsis; Biliary sepsis; Brucella sepsis; Burkholderia cepacia complex sepsis; Campylobacter sepsis; Candida sepsis; Capnocytophaga sepsis; Cerebral septic infarct; Citrobacter sepsis; Clostridial sepsis; Corynebacterium sepsis; Device related sepsis; Endotoxic shock; Enterobacter sepsis; Enterococcal sepsis; Erysipelothrix sepsis; Escherichia sepsis; Fungal sepsis; Group B streptococcus neonatal sepsis; Haemophilus sepsis; Helicobacter sepsis; Herpes sepsis; Herpes simplex sepsis; Intestinal sepsis; Klebsiella sepsis; Leclercia bacteraemia; Leptospira sepsis; Listeria sepsis; Meningococcal sepsis; Micrococcal sepsis; Myocarditis septic; Neutropenic sepsis; Nocardia sepsis; Pelvic sepsis; Plague sepsis; Pneumococcal sepsis; Porphyromonas bacteraemia; Post procedural sepsis; Postpartum sepsis; Pseudallescheria sepsis; Pseudomonal sepsis; Pulmonary sepsis; Salmonella sepsis; SARS-CoV-2 sepsis; Sepsis; Sepsis neonatal; Sepsis Pasteurella; Sepsis syndrome; Septic arthritis haemophilus; Septic arthritis neisserial; Septic arthritis staphylococcal; Septic arthritis streptobacillus; Septic arthritis streptococcal; Septic cerebral embolism; Septic coagulopathy; Septic embolus; Septic encephalopathy; Septic necrosis; Septic phlebitis; Septic pulmonary embolism; Septic rash; Septic shock; Septic vasculitis; Serratia sepsis; Shigella sepsis; Staphylococcal sepsis; Stenotrophomonas sepsis; Streptococcal sepsis; Toxic shock syndrome; Toxic shock syndrome staphylococcal; Toxic shock syndrome streptococcal; Umbilical sepsis; Urosepsis; Viral sepsis; Waterhouse-Friderichsen syndrome; Wound sepsis; Yersinia sepsis;

Part three NCCN guidelines related to ICIs' indications in this study

1.Bladder Cancer, Version 3.2020, NCCN Clinical Practice Guidelines in Oncology ;PMID: 32135513

2. Breast Cancer, Version 3.2020, NCCN Clinical Practice Guidelines in Oncology

PMID: 32259783

3. Colon Cancer, Version 2.2021, NCCN Clinical Practice Guidelines in Oncology

PMID: 33724754

4. Esophageal and Esophagogastric Junction Cancers, Version 2.2019, NCCN Clinical Practice Guidelines

in Oncology ; PMID: 31319389

5. Gastric Cancer, Version 2.2022, NCCN Clinical Practice Guidelines in Oncology

PMID: 35130500

6. Head and Neck Cancers, Version 2.2020, NCCN Clinical Practice Guidelines in Oncology ;PMID: 32634781

7. Hepatobiliary Cancers, Version 2.2021, NCCN Clinical Practice Guidelines in Oncology; PMID: 34030131

8. Hodgkin Lymphoma, Version 2.2020, NCCN Clinical Practice Guidelines in Oncology; PMID: 32502987

9. Melanoma, Version 2.2016, NCCN Clinical Practice Guidelines in Oncology PMID: 27059193

10. Merkel Cell Carcinoma, Version 1.2018, NCCN Clinical Practice Guidelines in Oncology; PMID: 29891526

11.NCCN Guidelines® Insights: B-Cell Lymphomas, Version 5.2021; PMID: 34781267

12. NCCN Guidelines Insights: Cervical Cancer, Version 1.2020; PMID: 32502976

13. NCCN Guidelines Insights: Kidney Cancer, Version 1.2021; PMID: 32886895

14. NCCN Guidelines Insights: Malignant Pleural Mesothelioma, Version 3.2016 PMID: 27407123

15. NCCN Guidelines® Insights: Melanoma: Cutaneous, Version 2.2021; PMID: 33845460

16. NCCN Guidelines Insights: Small Cell Lung Cancer, Version 2.2018; PMID: 30323087

17. NCCN Guidelines Insights: Uveal Melanoma, Version 1.2019 ;PMID: 32023525

18. NCCN Guidelines Updates: Management of Metastatic Colorectal Cancer PMID: 31117039

19.Uterine Neoplasms, Version 1.2018, NCCN Clinical Practice Guidelines in Oncology; PMID: 29439178

20. NCCN Guidelines Insights: Non-Small Cell Lung Cancer, Version 2.2021

PMID: 33668021

Detailed cancer regimens included to compare the signal of pulmonary sepsis in this study, please review the aers. load file.
